# Effects of prey density, temperature and predator diversity on nonconsumptive predator-driven mortality in a freshwater food web

**DOI:** 10.1038/s41598-017-17998-4

**Published:** 2017-12-22

**Authors:** Lukáš Veselý, David S. Boukal, Miloš Buřič, Pavel Kozák, Antonín Kouba, Arnaud Sentis

**Affiliations:** 1 University of South Bohemia in České Budějovice, Faculty of Fishery and Protection of Waters, South Bohemian Research Centre of Aquaculture and Biodiversity of Hydrocenoses, Zátiší 728/II, 389 25 Vodňany, Czech Republic; 2 University of South Bohemia, Faculty of Science, Department of Ecosystem Biology, Branišovská 1760, 370 05, České Budějovice, Czech Republic; 3 Czech Academy of Sciences, Biology Centre, Institute of Entomology, Laboratory of Aquatic Insects and Relict Ecosystems, Branišovská 1160/31, 370 05, České Budějovice, Czech Republic; 4 Unité Mixte de Recherche 5174 ‘Evolution et Diversité Biologique’, Université de Toulouse III - Institut de Recherche pour le Développement-Centre National de la Recherche Scientifique-École Nationale Supérieure de Formation de l’Enseignement Agricole. 118 route de Narbonne, F-31062 Toulouse, France

## Abstract

Nonconsumptive predator-driven mortality (NCM), defined as prey mortality due to predation that does not result in prey consumption, is an underestimated component of predator-prey interactions with possible implications for population dynamics and ecosystem functioning. However, the biotic and abiotic factors influencing this mortality component remain largely unexplored, leaving a gap in our understanding of the impacts of environmental change on ecological communities. We investigated the effects of temperature, prey density, and predator diversity and density on NCM in an aquatic food web module composed of dragonfly larvae (*Aeshna cyanea*) and marbled crayfish (*Procambarus fallax* f. *virginalis*) preying on common carp (*Cyprinus carpio*) fry. We found that NCM increased with prey density and depended on the functional diversity and density of the predator community. Warming significantly reduced NCM only in the dragonfly larvae but the magnitude depended on dragonfly larvae density. Our results indicate that energy transfer across trophic levels is more efficient due to lower NCM in functionally diverse predator communities, at lower resource densities and at higher temperatures. This suggests that environmental changes such as climate warming and reduced resource availability could increase the efficiency of energy transfer in food webs only if functionally diverse predator communities are conserved.

## Introduction

Investigating the effects of environmental drivers on food webs is crucial to better understand global change impacts on energy and nutrient fluxes across trophic levels. A growing number of studies have thus investigated the effects of global change drivers such as temperature, enrichment, pollutants, and habitat fragmentation on trophic interactions^1–4^. For example, previous studies have shown that predation rate often increases with temperature but decreases with prey density^5–8^. Thermal effects on predation rate are mainly driven by the acceleration of physiological processes (metabolism and digestion) leading to higher energetic demands of the predators, and by more frequent predator-prey encounters due to faster movement of predators or prey with warming. The effects of prey density are caused by the non-linearity of the predator feeding rate that increases with prey density and reaches a plateau at high prey densities (i.e., saturating Holling type II or III functional responses). Altogether, these temperature- and density-dependent effects on predation rates can alter population dynamics and species persistence by modifying trophic interaction strengths^9^.

However, prey also face predation-induced types of death other than direct consumption by predators. Predator attacks are not always successful and injured prey sometime escape and die later away from the predator^10,11^. Predators can also abandon or only partially consume some of the killed prey, a widespread behaviour in many invertebrate and vertebrate predators referred to as surplus killing^12–14^. This feeding behaviour is an important component of consumer-resource interactions that can influence population dynamics and predator-prey co-evolution^15–18^. Finally, the “ecology of fear” framework posits that the presence of predators can mobilize stress hormone secretion and consequently decrease prey energetic reserves^19,20^. Persistent stress reaction may thus “scare prey to death” and further increase prey mortality rates^21–24^. While surplus killing is well documented and relatively common, cases of prey mortality linked to high stress levels and unsuccessful predator attacks remain largely unexplored.

All these phenomena contribute to nonconsumptive predator-driven mortality (hereafter NCM) in food webs. Overall, the proportion of dead prey not eaten by predators can be substantial^11,25^. These prey individuals do not contribute to the flux of energy and nutrients to higher trophic levels, which can alter ecosystem functioning through lowered trophic transfer efficiency. Altogether, this suggests that NCM is relevant to the understanding and predictions of global change impacts on energy flux and ecosystem functioning.

Factors modulating NCM strength are insufficiently understood. Previous studies reported that prey availability strongly influences surplus killing^12,26–33^, which typically increases with prey density. Nevertheless, the dependence of surplus killing on prey density varies strongly among taxa and can be linear or unimodal^34–36^. Moreover, we are not aware of any study about prey density effects on prey mortality linked to high stress levels caused by predation risk.

The role of global change drivers in NCM is essentially unknown. Human activities lead to rapid environmental changes including pollution, habitat alteration, nutrient enrichment, and global warming. Understanding how these drivers impact organisms and their interactions, including surplus killing and other processes affecting energy transfer across trophic levels, is important to better predict global change consequences on Earth’s biota^4,37–40^. To our knowledge, the effects of temperature (or any other of the above drivers) on the “prey scared to death” phenomenon and unsuccessful predator attacks remain unexplored. Only one study reported a decrease in surplus killing with warming^34^, possibly due to higher metabolic demands of predators at warmer temperatures and hence higher ingestion rates required to fulfil these demands^23^.

Most food webs consist of multiple predators that share similar prey^14,41^ and provide important ecosystem services^42,43^. There is mounting evidence that the effects of multiple predators on prey populations can rarely be predicted from single-predator effects. Interactions among multiple predators and their prey often result in emergent effects such as predation risk reduction or enhancement^6,44–46^. Does this disconnect between observations based on single and multiple predators also apply to NCM? Benke^47^ suggested that interactions among conspecific predators increase surplus killing, which could in turn exacerbate the effects of exploitative competition among conspecific predators^48^. However, no study has compared prey surplus killing in intraspecific and interspecific predator assemblages, which limits our knowledge about the relative importance of the effects of predator density versus diversity on surplus killing.

The impact of multiple predators and functionally diverse predator communities on the amount of prey “scared to death” has not been thoroughly explored either. More generally, knowledge of the impact of multiple predators and predator functional diversity on NCM are limited. Previous studies reported that predator diet breadth and functional diversity within predator assemblages can strongly affect the relationship between predator diversity and ecosystem functioning (e.g. primary production and prey suppression via trophic cascades)^49,50^. For instance, Finke and Snyder^51^ suggested that communities composed of generalist consumers exploit resources better than those including only specialists. Similarly, communities including diverse consumer types such as predators, omnivores and scavengers should exhibit reduced NCM values compared to communities composed only of predators^24^, e.g., when scavengers and omnivores eat prey killed by other predators. These observations provide qualitative insights but do not sufficiently advance our ability to quantify NCM strengths in predator-rich communities. In this study, we experimentally investigated the effects of temperature, resource availability (i.e., prey density), and predator density and diversity on NCM strengths (i.e., the proportion of dead prey not eaten by predators). Changes in temperature and resource availability are two of the most important global change drivers^7^. It is thus crucial to investigate their impacts on energy fluxes to better understand the consequences of global change on ecological communities^6,52^. Our study provides an initial step in the exploration of the effects of abiotic and biotic factors on NCM strengths in food webs. It helps better understand and predict global change consequences on energy fluxes in ecological communities.

## Results

While prey mortality was negligible in controls without predators (0–2% of the initial prey died during the experiment, mean ± SD = 0.84 ± 1.06%), the proportion of dead uneaten prey per predator (see Methods for details; hereafter only NCM strength) was significantly positive in treatments with predators. The overall average value of NCM in treatments with predators was 4% with a maximum of 20%. Moreover, we found dead uneaten prey at all prey densities as well as in each predator assemblage. In addition, dead uneaten prey were found in 64% of the replicates with predators.

**Figure 1:**
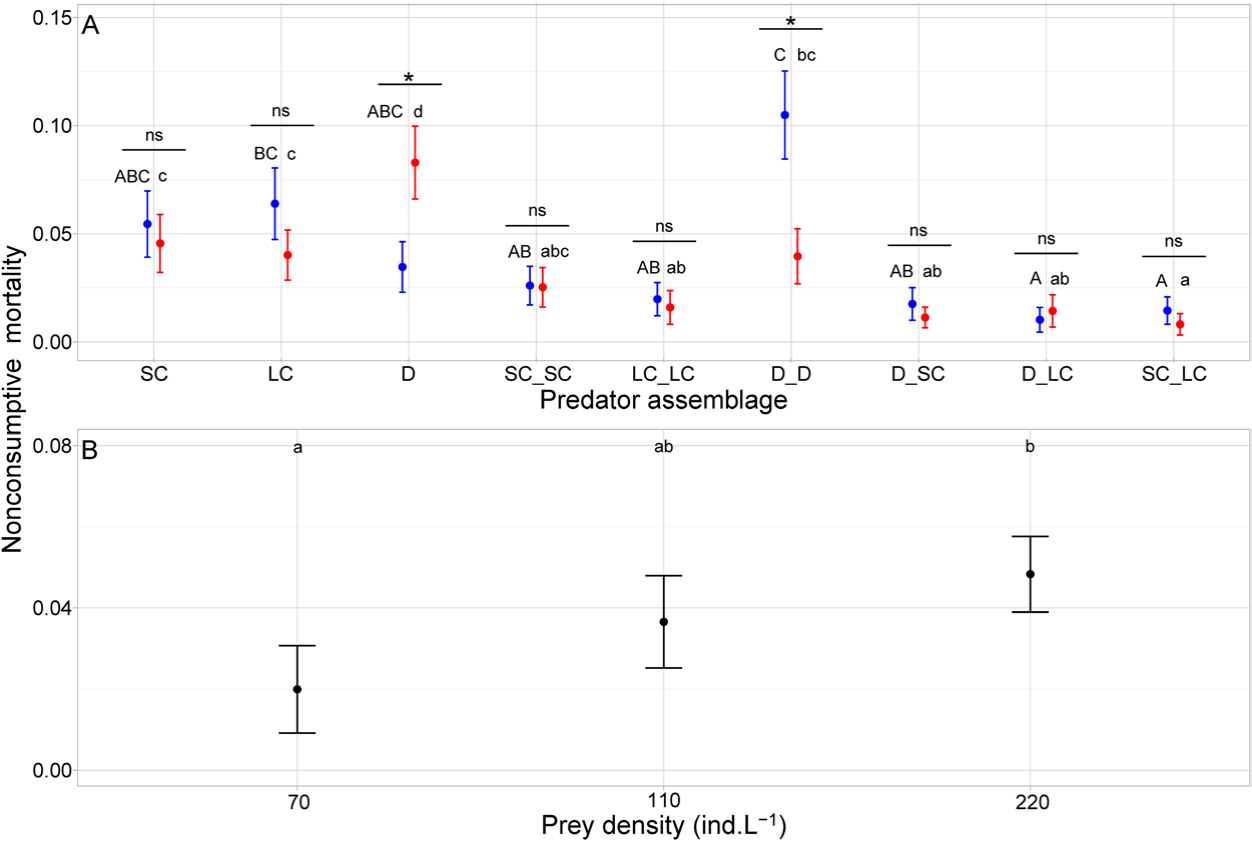
Dependence of *per capita* NCM strengths (number of dead uneaten prey per predator; mean ± 95% CI) on temperature, prey density and predator assemblage. (**A**) NCM strengths for all predator treatments and prey densities at 16 °C (blue) and 20 °C (red). Predator assemblages: D = dragonfly larva, SC = small crayfish, LC = large crayfish; predator pairs with underscore. Significant differences (P < 0.05) between temperatures within each predator assemblage marked by asterisk; ‘ns’ = differences not significant. Significant differences (P < 0.05) between predator assemblages at given temperature marked with different letters (16 °C = capital letters, 20 °C = small letters). (**B**) Effect of prey density on NCM strength across all predator assemblages and both temperatures.

**Figure 2:**
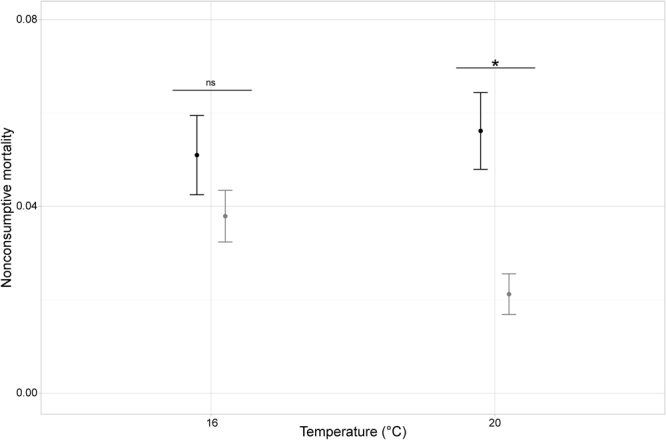
Dependence of *per capita* NCM strength (mean ± 95% CI) on predator density at two temperatures. Black bars = single predator treatments (D, SC and LC), grey bars = predator pair treatments (D_D, SC_SC and LC_LC). Dependence on prey identity (not shown) qualitatively identical to those shown in Figs 1B and 3B.

NCM strength varied significantly with temperature, prey density and predator assemblage (Table 1). Furthermore, temperature effect depended on predator assemblage (significant temperature × predator assemblage interaction, Table 1 and Fig. 1). Warming decreased the strength of NCM caused by dragonfly pairs, but had the opposite effect in the single dragonfly treatment and did not affect NCM strength in the other predator treatments (Fig. 1A). NCM strength increased significantly with prey density (Fig. 1B) and this effect was independent of temperature or predator treatments (Table 1). In addition, we found a temperature-dependent effect of predator density on NCM strength that was independent of prey density and predator species and size (Table 2). NCM strength tended to decrease with predator density, but this effect was more pronounced and significant only at 20 °C (Table 2 and Fig. 2).

**Table 1:** F and *p* values of the most parsimonious GLM for the effects of temperature, prey density and predator assemblage on NCM strength.

	df	resid. df	F	*p*
temperature	1	446	3.23	0.07
prey density	1	445	21.4	<**0.001**
predator assemblage	8	437	16.6	<**0.001**
temperature × predator assemblage	8	429	3.82	<**0.001**

Data were corrected for predator density by dividing the proportion of dead prey not eaten by predator density. df = degrees of freedom, resid. df = residual degrees of freedom. Bold values represent significant explanatory variables (*P* < 0.05).

**Table 2:** F and *p* values of the most parsimonious GLM for the effects of temperature, prey density, and predator identity and density on *per capita* NCM strength.

	df	resid. df	F	*p*
temperature	1	343	2.02	0.24
prey density	1	342	10.81	**0.002**
predator density	1	341	21.31	**0.002**
temperature × predator density	1	340	9.03	**0.005**

Only single predator treatments and treatments with predator pairs of the same size and species were used in this analysis. Variables not retained in the final model are omitted. Symbols as in Table 1.

**Figure 3:**
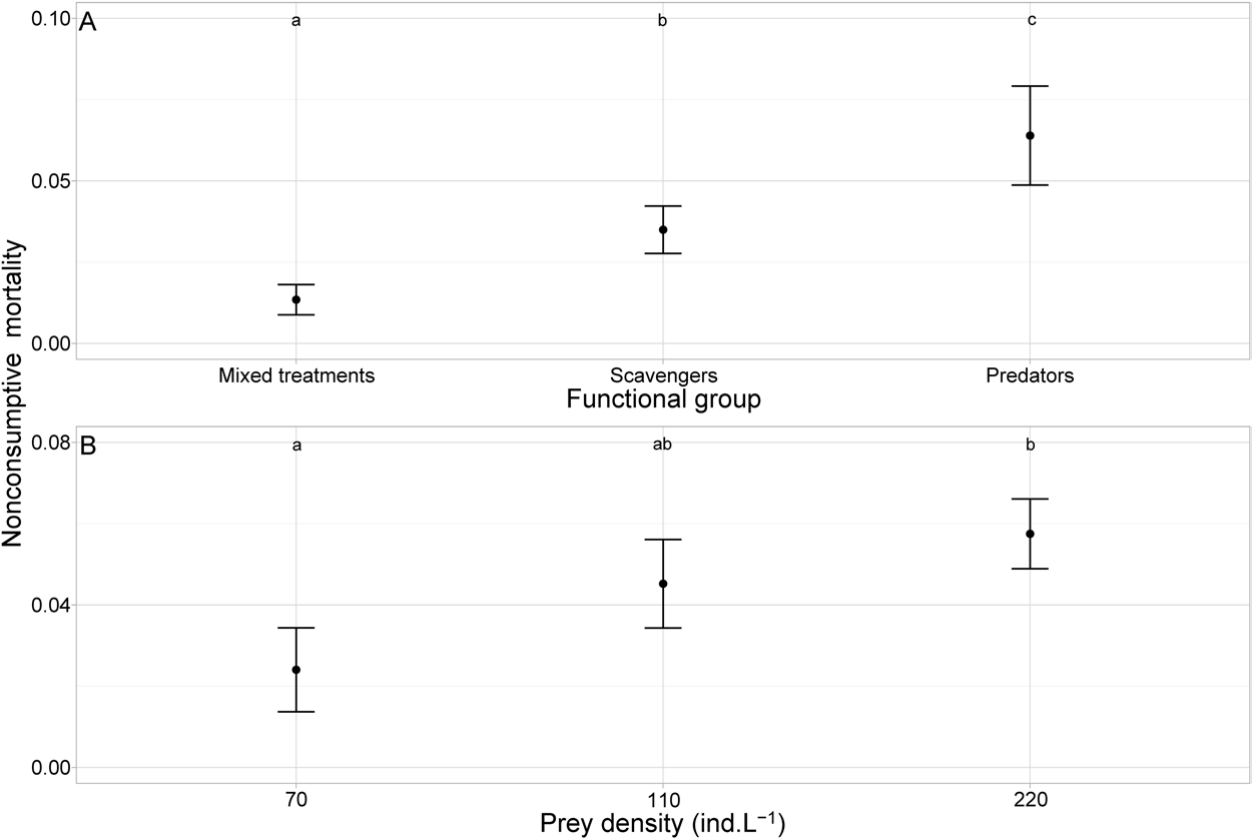
Dependence of *per capita* NCM strength (mean ± 95% CI) on prey density and predator functional groups. (**A**) NCM strength for each functional group. Mixed treatments (i.e., scavenger and predator) = D_SC and D_LC; scavengers = SC, LC, SC_SC, LC_LC and SC_LC; predators = D_D and D. (**B**) Effect of prey density on NCM strength. Different letters mark significant differences (P < 0.05) between predator functional groups or prey density.

When grouping the treatments by predator functional groups (predators, scavengers and their mix), we found that NCM strength varied significantly among functional groups and with prey density but did not depend on temperature or any statistical interactions of these three variables (Table 3). NCM was lowest in mixed treatments involving one scavenger and one predator and highest in treatments involving only predators (Table 3 and Fig. 3). The estimated dependence of NCM strength on prey density was nearly the same as when we grouped the data by predator assemblages (compare Figs 1B and 3B).

**Table 3:** F and *p* values of the most parsimonious GLM for the effects of temperature, prey density and predator functional group on *per capita* NCM strength.

	df	resid. df	F	*p*
prey density	1	445	20.2	<**0.001**
functional group	2	444	37.7	<**0.001**

Variables not retained in the final model are omitted. Symbols as in Table 1.

**Figure 4:**
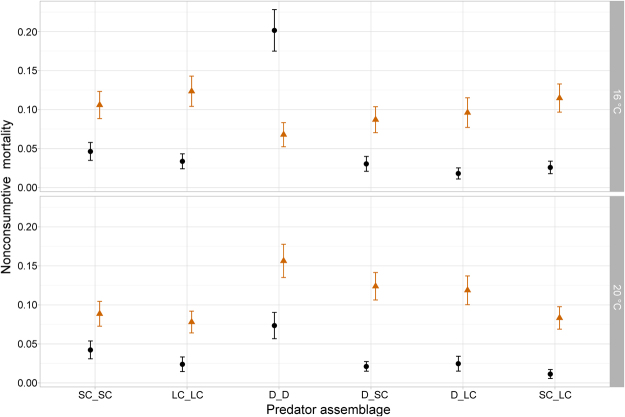
Comparison of the observed and predicted NCM strengths (mean ± 95% CI) for each temperature and predator pair averaged over all prey densities. Data were not corrected for predator density. Black bars and circles = observed values; orange bars and triangles = predicted values were generated using multiplicative risk model.

Predictions from the multiplicative risk model mostly overestimated the observed NCM strengths except for the treatment with two dragonflies at 16 °C, in which the observation exceeded the prediction (Fig. 4). That is, NCM strengths in predator assemblages were almost always weaker than expected from single-predator treatments.

Temperature, prey density and predator assemblages and density affected the *per capita* proportions of dead prey with and without visible attack marks in very similar but not identical ways to how they affected the overall NCM strength. Both *per capita* proportions were significantly affected by temperature, prey density and predator assemblage, and the statistical interaction between temperature and predator assemblage (Table S1). Warming significantly reduced the *per capita* proportion of dead prey with visible attack marks in two intraspecific predator assemblages (D_D and LC_LC) but had no effect in the other assemblages (Table S1 and Fig. S1). Warming also reduced the *per capita* proportion of dead prey without visible attack marks in the D_D assemblage but had the opposite effects in the single dragonfly treatment (Table S2 and Fig. S2). Finally, the *per capita* proportions of both types of dead prey increased with initial prey density (Figs S1B and S2B).

## Discussion

Nonconsumptive predator-driven mortality (NCM) is a common but underestimated component of predator-prey interactions. Previous studies mainly focused on consumptive mortality and often neglected NCM linked to unsuccessful predator attacks, surplus killing or predator-induced high stress levels. These three different sources of mortality are widespread across many invertebrate and vertebrate taxa^12,23,24,34,35,53,54^ and can influence population dynamics, food web structure, and the co-evolution of predators and prey^16,17,34^. However, the magnitude and dependence of NCM on external factors remains largely unexplored, which limits our understanding of when and how biotic and abiotic factors influence the strength of consumer-resource interactions and thus the dynamics and structure of ecological communities. Here, we investigated the effects of temperature, prey density, and predator density and functional diversity on NCM strengths in an aquatic food web module.

Like all laboratory studies, our experiments have limitations that prevent strong quantitative inference from the results. They were conducted in an artificial environment at a small spatio-temporal scale that cannot be directly extrapolated to long-term community and ecosystem dynamics. The environment and arena size could have influenced prey mortality, but we found it to be negligible in control trials without predators and much lower than the observed magnitude of NCM in predation trials. This suggests that the qualitative patterns found in our experiment are sufficiently robust. Moreover, the habitat volume and duration of our experimental trials fall within the range commonly used in predation experiments with aquatic invertebrate predators. We therefore think that our study helps identify factors influencing NCM strengths and provides an additional step towards a better understanding of the effects of biotic and abiotic factors on predator-prey interaction strengths in aquatic systems.

### Effect of temperature on NCM

We found that NCM strength was not influenced by temperature except in treatments involving only dragonflies. This indicates that the effect of temperature on NCM is species specific and potentially related to consumer functional type (pure predator vs. scavenger). Moreover, the effects of temperature depended on dragonfly density: warming increased NCM in treatments with a single dragonfly whereas it decreased NCM in treatments with two dragonflies. Our more detailed analyses revealed that the effect of temperature in the single dragonfly treatment was caused by a magnified “scared to death” phenomenon rather than a change in surplus killing. Although the mechanisms and physiological processes underlying these effects remain to be investigated in more detail, our results suggest that the additional stressor (i.e., warming) led to increased mortality of fish fry in the presence of a single dragonfly predator.

As the *per capita* prey density was reduced in treatments with two dragonfly predators, it is plausible that they fed on the prey more efficiently to compensate for the joint effect of higher metabolic demands and lower prey availability at the higher temperature. Additional aspects of predator and prey behaviour that would alter NCM strength with temperature may also change with predator density. For instance, predators can become more careful when catching and handling prey and hence feed more efficiently in the presence of other predators^14,43,44^, and their awareness of other predators may increase with temperature due to more frequent mutual encounters. Overall, our results indicate that warming effects on NCM strengths depend on predator identity and density, which we discuss next in more detail. It is currently difficult to generalize our findings given the paucity of studies on this topic. We thus call for further studies investigating the effects of temperature on NCM strength in food webs.

### Effect of prey density on NCM

NCM strength significantly increased with prey density and this effect was independent of temperature and predator identity or density. Prey density effect on overall NCM strength was driven by a combined increase of surplus killing and “scared-to-death” mortality with prey density. Previous studies have also show that surplus killing is more frequent at higher prey densities^12,34,35,55^, but the shape of this relationship varies among taxa from linear to unimodal. We found a nearly linear relationship between prey density and surplus killing, which corroborates the results of previous studies on predatory aquatic insects including larvae of the damselfly *Anomalagrion hastatum* ^56^, aquatic bug *Diplonychus rusticus* ^27^ and the backswimmer *Notonecta hoffmanni* ^36^.

To our knowledge, the effect of prey density on nonconsumptive predator-induced mortality has never been explored. We found that, while prey mortality in the absence of predators was negligible and did not increase with prey density, the proportion of dead prey without visible attack marks increased strongly with prey density, suggesting that prey are more “scared to death” by predators in denser prey populations. Higher stress levels in the prey may result from oxygen depletion or more frequent physical contacts with conspecifics. These stressors alone may be sublethal but can become lethal when magnified by or combined with an additional stressor such as predator presence^57,58^. For instance, predators can increase prey respiration rate (e.g., if predator avoidance requires faster or more frequent swimming), which would accelerate oxygen depletion and increase prey mortality. This effect is likely to be stronger at high prey densities when prey are more likely to deplete oxygen. Although we cannot resolve the mechanism underlying the “scared to death” phenomenon in our experiment, our results indicate that predator presence can modify this type of prey mortality. Interestingly, the effects of prey density on “scared-to-death” mortality and surplus killing were independent of predator species and assemblage, suggesting a general effect of prey density on NCM strengths. We thus predict that declines in trophic transfer efficiency due to NCM will become more pronounced at higher prey densities. This would act as a stabilizing factor in communities with fluctuating predator and prey population densities^59^.

### Effects of predator density and functional diversity on NCM

The observed decline in *per capita* NCM with predator density, especially at the higher temperature, can be explained by a combination of two behavioural responses: increased individual feeding rates and the ability of predators to recognize conspecifics. The former response would help cover higher metabolic demands of predators at warmer temperatures^23^, while the latter would enable them to adjust to the perceived scarcity of resources. Further investigations are needed to determine which of these two behavioural responses contributes most to the observed pattern.

Interactions among predators and predator functional types can strongly influence consumer-resource interactions in species-rich communities^6,24,60,61^. We found that NCM strength varied substantially among predator assemblages, being higher in pure predators (i.e., dragonflies) than in scavengers (i.e., crayfish). Interestingly, NCM strength was lowest when a predator and a scavenger were paired together. The underlying mechanisms remain to be investigated in more detail. We assume that scavengers either feed on the dead prey abandoned by dragonfly larvae that cannot locate immobile prey^62^ or that scavengers and predators modify their behaviour when together. Whatever the exact mechanism, our results suggest that increased predator functional diversity in food webs can lower NCM strengths.

Moreover, multi-predator NCM strengths in our experiment could not be predicted from single-predator NCM strengths alone. Both predator density and predator diversity, including the functional differences between pure predators (dragonfly larvae) and scavengers (crayfish), thus affected NCM strength. Overall, our results suggest that trophic transfer efficiency is higher in functionally diverse ecosystems, which may have important implications for population dynamics and community structure.

## Conclusions

Nonconsumptive mortality is an important but under-appreciated component of consumer-resource interactions. Here we showed that abiotic and biotic factors such as temperature, prey density, predator functional diversity and density influence NCM strength. The effect of temperature on NCM strength varied among predator assemblages and was often not significant. On the other hand, NCM strength increased with prey density independently of temperature and predator assemblage, suggesting a general effect of prey density on NCM strength. Moreover, NCM strength declined in functionally diverse predator assemblages. Our results indicate that energy transfer across trophic levels is more efficient in functionally diverse predator communities, at lower resource densities and at higher temperatures, which has important implications for community dynamics, ecosystem services, and biological conservation.

## Material and Methods

Experiments were conducted at the Research Institute of Fish Culture and Hydrobiology in Vodňany (RIFCH), Czech Republic during summer 2015. No specific permissions were required for capturing and manipulating the organisms used in the experiments. The study did not involve endangered or protected species. All experimental manipulations (capture, rearing and measurements) followed principles of animal welfare and their protection against abuse. We used two size classes of marbled crayfish *Procambarus fallax* f. *virginalis* (Decapoda; Cambaridae) and one size class of the dragonfly *Aeshna cyanea* (Odonata; Aeshnidae) as predators preying on common carp *Cyprinus carpio* (Cypriniformes; Cyprinidae) fry in the protopterygiolarval ontogenetic phase^55^. Marbled crayfish is an actively searching, benthic omnivore that is currently invading most freshwater ecosystems in Europe^63^. Larvae of the dragonfly *Aesha cyanea* are widespread native predators that can alternate between a ‘sit-and-wait’ and active foraging strategy targeting moving prey, and are often top predators in small fishless water bodies^62^.

Dragonfly larvae were collected in small sandpit pools in southern Bohemia and released back to the source locality after the experiments. Fish fry were obtained from a hatchery belonging to RIFCH. Crayfish were obtained from laboratory cultures maintained at RIFCH. Before the experiment, predators and prey were maintained at 16 °C and respectively fed in excess with sludge worm (*Tubifex tubifex*) and brine shrimp (*Artemia salina*) nauplii. Dragonfly larvae were maintained individually in 0.5-litre plastic boxes (125 × 45 × 80 mm) with 0.4 litres of aged tap water containing a willow twig as a perching site. Crayfish were kept in groups at low densities (0.8 ind.L^−1^) in 50-litre aquaria with access to shelters (>1 per animal) to avoid excessive competition and cannibalism.

### Experimental design

We standardized prey size (mean total length ± SD: 6.42 ± 0.20 mm) and used F-1 instar dragonfly larvae (further abbreviated as D) (total length: 30.1 ± 2.3 mm, wet weight: 0.53 ± 0.12 g) and two sizes of crayfish: small (abbreviated as SC; mean carapace length: 11.3 ± 0.9 mm, measured from the tip of the rostrum to the posterior edge of cephalothorax; wet weight: 0.45 ± 0.13 g) and large (LC; mean carapace length: 15.5 ± 1.0 mm; wet weight: 1.12 ± 0.18 g). One day before the experiment, predators were placed individually without food in 0.5-litre plastic boxes (125 × 45 × 80 mm) filled with 0.4 litres of aged tap water. Four hours before the experiment, predators were acclimated to the experimental temperature (16 or 20 °C). Similarly, prey were acclimated to the experimental temperature four hours before the experiment and were kept in 20-litre buckets. Experimental arenas consisted of plastic boxes (163 × 118 × 62 mm) filled with 1 litre of aged tap water and lined with a 1 cm layer of fine crystalline sand.

We performed a full factorial experiment with two temperature regimes (16 and 20 °C), three prey densities (70, 110, 220 ind. L^−1^, representing low, medium, and high prey densities based on pilot experiments), and nine predator treatments with the three predator types: single predators (3 treatments), pairs with two predators of the same size and species (3 treatments), and pairs with two predators differing in size or species (3 treatments). Each combination of temperature, prey density and predator treatment was replicated seven times. In addition, five controls without predators were deployed to assess background mortality of prey for each combination of temperature and prey density.

Prey were introduced into the experimental arenas for acclimation one hour before the start of the experiment. All predators were simultaneously introduced into the experimental arenas at the start of the experiment. After 24 hours, predators were removed and the number of living, killed (with visible attack marks), and dead prey (without visible attack marks) were recorded. During a pilot experiment, we observed that prey killed by the predators used in this study always had visible attack marks and all predator attacks were successful. Moreover, we did not observe partially eaten prey in this experiment. Although we did not directly measure stress levels of the fish fry, we attribute dead prey without visible attack marks to mortality due to high stress levels associated with predator presence.

### Statistical analyses

Prey mortality in controls without predators was negligible (range 0–2% of initial prey) and prey mortality in controls without predators did not increase with prey density (GLM, F_1,68_ = 2.07, *p* = 0.15). The data were thus not corrected for background mortality. We calculated *per capita* NCM strength as the ratio of dead uneaten prey density over initial prey density, divided by the number of predators. In addition, we also calculated an alternative measure of *per capita* NCM strength as the ratio of the density of dead uneaten prey over the density of eaten prey, divided by the number of predators. As the results were qualitatively similar, we do not present results for the latter NCM metric. We also tested the goodness of fit of our models using the Hosmer-Lemeshow test and verified that all models fitted the data well (*P* > 0.05). All model results are shown as mean ± 95% Wald confidence interval (CI).

We tested whether the *per capita* NCM strength (hereafter only NCM strength) is influenced by temperature, prey density, predator assemblage and their interactions using a GLM with a quasibinomial distribution to account for overdispersion^64^. The most parsimonious model was determined by sequential deletion of the least significant explanatory parameters or interaction terms from the full model. Parameter significance was evaluated using F-tests from the analysis of deviance. The final model included only parameters with significant p-values, and post-hoc Tukey tests were used to assess significant differences among treatment means. Finally, we grouped predator assemblages by functional groups: predators (i.e., only dragonfly larvae), scavengers (i.e., only crayfish), and mixed treatment (one scavenger and one predator) and analysed the effect of temperature, prey density and functional group on NCM strength as described above.

We tested whether NCM strength in multiple-predator assemblages can be predicted using our experimental data from single predator treatments. For this purpose, we used the multiplicative risk model that often appears in studies investigating predation rate by multiple predators on a single prey species^65^:

1 $$N{C}_{ab}={N}_{p}({P}_{a}+{P}_{b}-{P}_{a}{P}_{b})$$

where *NC*_ab_ is the predicted NCM strength measured as the density of dead uneaten prey, *N*_p_ is the initial prey density, and *P*_a_ and *P*_b_ are NCM strengths measured as the respective proportions of dead uneaten prey in single predator *a* and *b* treatments.

To better understand the mechanisms underlying our results, we further tested the influence of temperature, prey density, predator assemblage and their interactions on the *per capita* (i.e., per predator) proportion of dead prey with and without visible attack marks using two GLMs (one for each dependent variable) with quasibinomial distribution. The most parsimonious model was determined by sequential deletion of the least significant explanatory parameters or interaction terms from the full model and parameter significance was evaluated using F-tests from the analysis of deviance.

Finally, we tested whether *per capita* NCM strength depended on predator density along with predator identity, temperature, prey density and their interactions. Only single predator treatments and treatments with predator pairs of the same size and species were used in this analysis. We again used GLMs (one for each dependent variable) with quasibinomial distribution and proceeded with model selection and evaluation of parameter significance as above. All analyses were implemented in R version 3.2.5^66^.

### Data availability

Primary data used in this study are available on Dryad repository server.

## Electronic supplementary material

Supplementary information
