# Supplementary material for: Effects of prey density, temperature and predator diversity on nonconsumptive predator-driven mortality in a freshwater food web
Source: Sci Rep. 2017 Dec 22;7:18075. doi: 10.1038/s41598-017-17998-4 (PMC5741715; doi:10.1038/s41598-017-17998-4)

**Effects of prey density, temperature and predator diversity on nonconsumptive predator-driven mortality in a freshwater food web**

Lukáš Veselý^1*^, David S. Boukal^2,3^, Miloš Buřič^1^, Pavel Kozák^1^, Antonín Kouba^1^ and Arnaud Sentis^2,3,4^

*^1^University of South Bohemia in České Budějovice, Faculty of Fishery and Protection of Waters, South Bohemian Research Centre of Aquaculture and Biodiversity of Hydrocenoses, Zátiší 728/II, 389 25 Vodňany, Czech Republic*

*^2^University of South Bohemia, Faculty of Science, Department of Ecosystem Biology, Branišovská 1760, 370 05 České Budějovice, Czech Republic*

*^3^ Czech Academy of Sciences, Biology Centre, Institute of Entomology, Laboratory of Aquatic Insects and Relict Ecosystems, Branišovská 1160/31, 370 05 České Budějovice, Czech Republic*

*^4^Unité Mixte de Recherche 5174 ‘Evolution et Diversité Biologique’, Université de Toulouse III- Institut de Recherche pour le Développement*-*Centre National de la Recherche Scientifique-*École Nationale Supérieure de Formation de l’Enseignement Agricole*. 118 route de Narbonne, F-31062 Toulouse, France.*

*Corresponding author. Tel.: +420 389 034 745

*E-mail address*: [veselyl@frov.jcu.cz](mailto:veselyl@frov.jcu.cz) (L. Veselý)

**Table S1**. F and *p* values of GLM of the dependence of the *per capita* proportion of dead prey with visible attack marks per predator on temperature, prey density and predator assemblage. df = degrees of freedom, resid. df = residual degrees of freedom. Bold values indicate significant explanatory variables (*P* < 0.05).

|  | df | resid. df | F | *p* |
| --- | --- | --- | --- | --- |
| temperature | 1 | 446 | 7.67 | **0.005** |
| prey density | 1 | 445 | 9.47 | **0.002** |
| predator assemblage | 8 | 437 | 16.56 | **< 0.001** |
| temperature x predator assemblage | 8 | 429 | 3.89 | **< 0.001** |

**Table S2**. F and *p* values of GLM of the dependence of the *per capita* proportion of dead prey without visible attack marks per predator on temperature, prey density and predator assemblage. Symbols as in Table S1.

|  | df | resid. df | F | *p* |
| --- | --- | --- | --- | --- |
| temperature | 1 | 446 | 1.21 | 0.27 |
| prey density | 1 | 445 | 2.92 | **< 0.001** |
| predator assemblage | 8 | 437 | 16.56 | **< 0.001** |
| temperature x predator assemblage | 8 | 429 | 3.89 | **< 0.001** |

**Figure S1.** Dependence of the *per capita* proportion of dead prey with visible attack marks (mean ± 95% CI) on temperature, prey density and predator assemblage. (A) Dependence on temperature for all predator treatments and prey densities at 16°C (blue symbols) and 20°C (red symbols). (B) Dependence on initial prey density. Predator assemblages and other symbols as in Fig. 1.


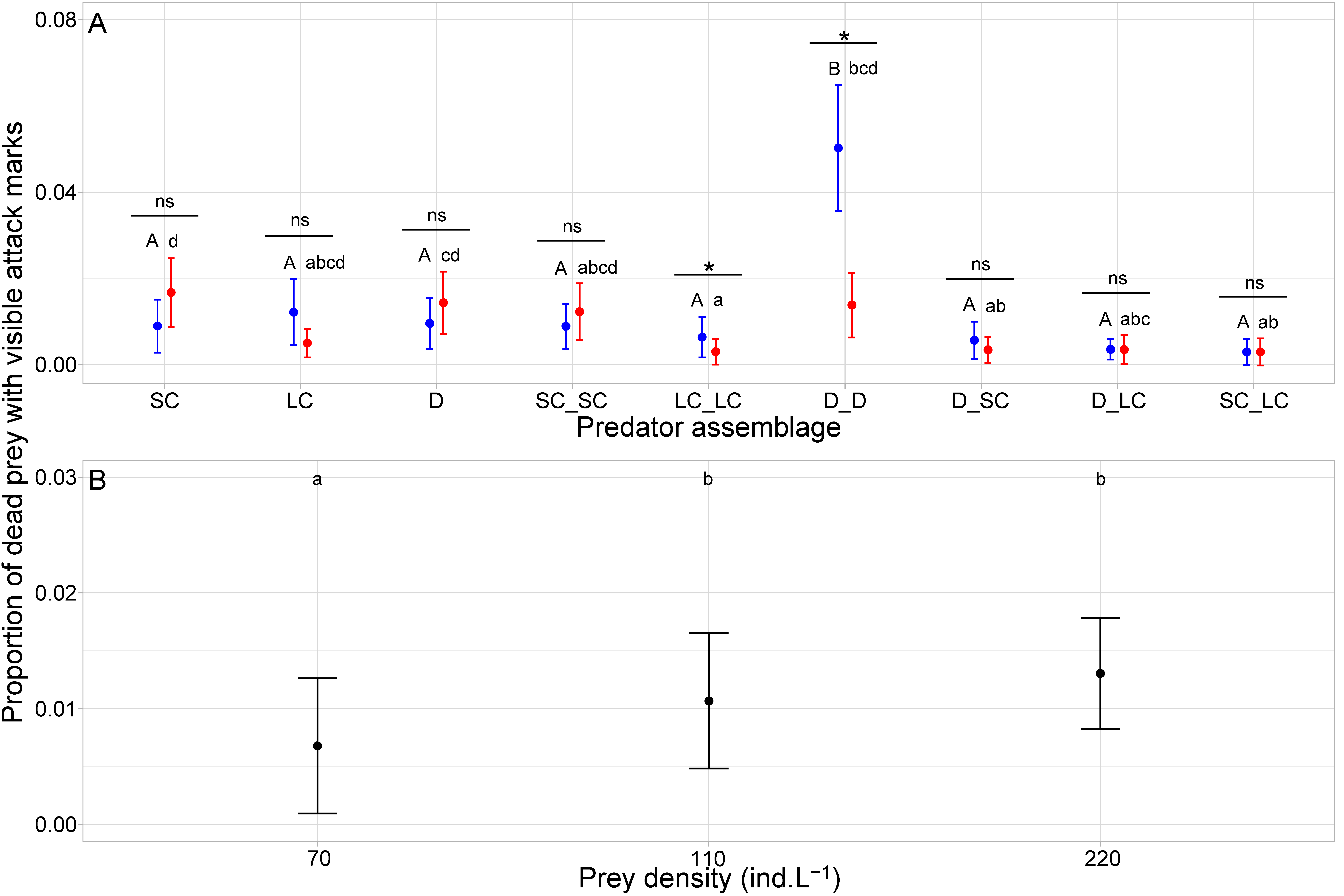


**Figure S2** Dependence of the *per capita* dead prey without visible attack marks on temperature, prey density and predator assemblage. Data shown as mean ± 95% CI (per capita). (A) Dependence on temperature for all predator treatments and prey densities at both temperatures. (B) Dependence on initial prey density. Symbols and letters as in Fig. 1.


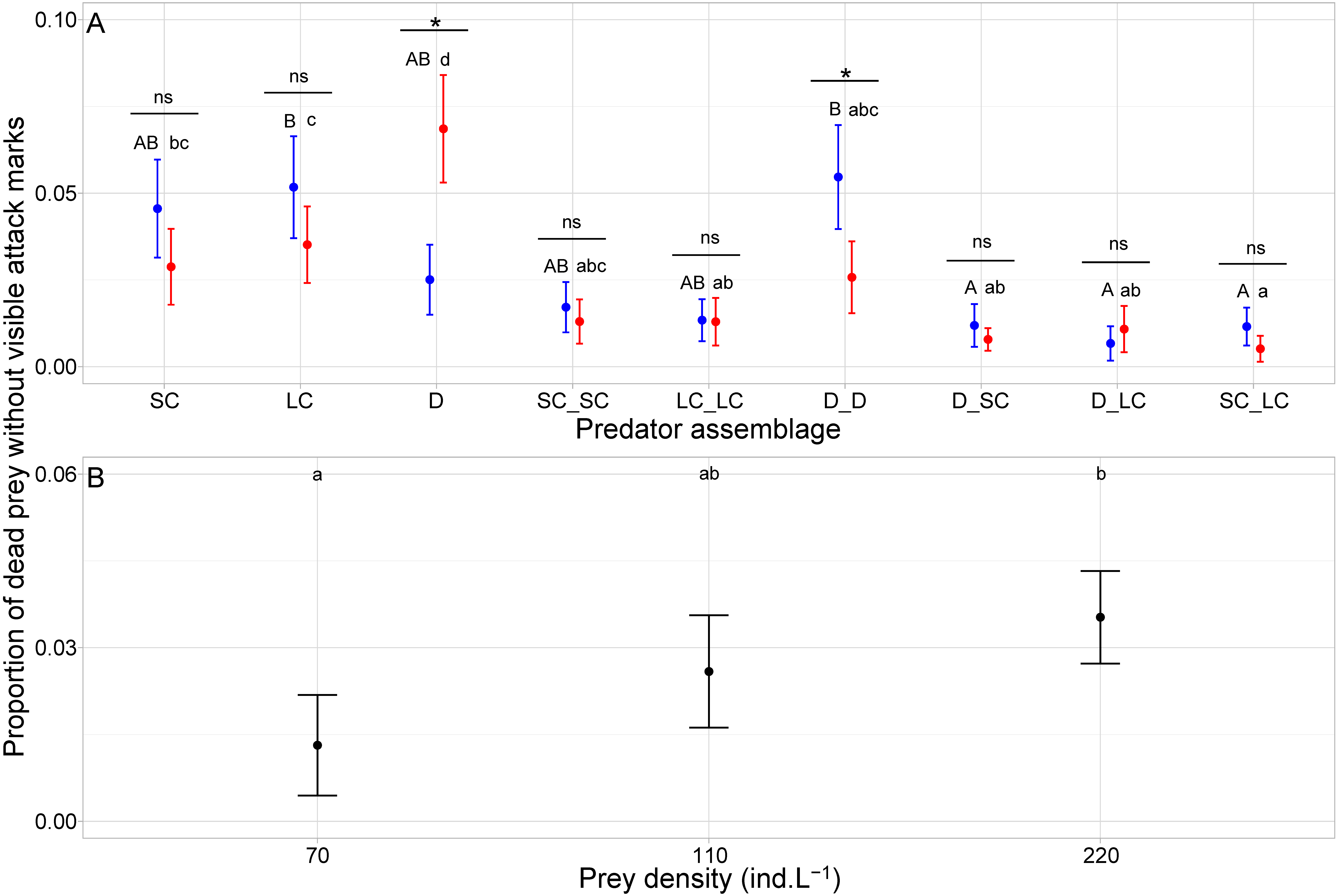

Supplement: Supplementary file 1 — Supplementary information [file 41598_2017_17998_MOESM1_ESM.doc]
